# Professor Swing on Immortality

**Published:** 1891-02

**Authors:** 


### PROFESSOR SWING ON IMMORTALITY.

To believe well in a future beyond, it seems essential that one make the assumption of spirit a starting point, and then the whole material world becomes its servant, or its arena, or decoration; but if, with Huxley and Darwin, we begin with the assumption of matter, there seems nothing to throw us over across the dividing ocean, and we must remain on the shore of dust, and hence death ; for, move to and fro as material does from wild rose to full leaved rose, from ape to man, it always brings us at last only the dust. There is no immortal rose, however full leaved it may become. Death is its destiny. To get over this tomb of roses and of man it is essential that a spirit be assumed; a God, an essence differing from the vital action of the heart or of the roots of the wild flowers. In this study of man, after we assume that he possesses a spirit, the text enters with its single thought that God is not a God of dead souls, but of living ones. There is no manifest reason for supposing a soul made in such a divine image to be only an ephemeral creature, going quickly to nothingness, thus making God the father of the dead rather than of the living. All the reasons for creating such a being as man remain for continuing his existence. If, when the Creator had formed such a universe as lies around us here, of which our system is as a grain of sand upon an infinite shore, He finally concluded to make man a race to inhabit one or more stars of the universe, a race in the divine image, a human life of a few years would seem wholly unworthy of such a boundless material realm ; for we cannot master its truths nor taste happiness in any threescore-years career. Your children have shown their divine nature, have spoken a few words, have rejoiced in a few springtimes, and have gone hence, leaving you heartbroken. A brief career is thus not in harmony with the immense universe in which this life begins, and of which man is unquestionably the highest order of beings.—*American Spectator*.

